# Serum nitric oxide levels in healthy pregnant women: a case- control study in a tertiary facility in Ghana

**DOI:** 10.1186/s40748-017-0072-y

**Published:** 2018-02-20

**Authors:** Ebenezer Owusu Darkwa, Robert Djagbletey, Daniel Sottie, Christian Owoo, Naa Martekuor Vanderpuye, Raymond Essuman, George Aryee

**Affiliations:** 1 Department of Anaesthesia, Korle-bu Teaching Hospital, University of Ghana School of Medicine and Dentistry, College of Health Sciences, P. O. Box 4236, Accra, Ghana; 2 Department of Anaesthesia, Korle-Bu Teaching Hospital, Accra, Ghana

**Keywords:** Healthy pregnancy, Serum nitric oxide, Non-pregnant, Blood pressure

## Abstract

**Background:**

Pregnancy is associated with significant changes in maternal cardiovascular system which regulates oxygen and nutrient supply to the growing foetus. Nitric oxide, a physiologic vascular smooth muscle relaxant regulates blood flow and therefore may play a role in the cardiovascular changes in pregnancy. The study aimed to determine the levels and changes in maternal serum nitric oxide levels during healthy pregnancy.

**Methods:**

A case-control study was conducted among 32 healthy non-pregnant women as controls and 100 healthy pregnant women (consisting of 33 first trimester, 37 s trimester, and 30 third trimester) as cases. Subjects were consecutively recruited into the study after obtaining an informed consent and meeting the inclusion criteria. Griess Reagent method was used to determine serum nitric oxide levels.

**Results:**

There were no statistically significant difference in the ages and parity of recruited cases and controls. Mean arterial blood pressures were significantly lower (*p* = 0.009) and serum nitric oxide levels were significantly higher (*p* < 0.001) in healthy pregnant women compared to healthy non-pregnant women. There was a non-significant progressive increase in serum nitric oxide levels during healthy normal pregnancy.

**Conclusions:**

The finding of a significantly reduced blood pressures and a significant increase in serum nitric oxide levels in healthy pregnancy may suggest a role of nitric oxide in vascular adaptation in pregnancy.

## Background

Numerous changes in the cardiovascular system occur during normal pregnancy to reduce maternal vascular resistance thereby increasing maternal oxygen delivery which is necessary to meet the increased metabolic demand and higher oxygen consumption during pregnancy [1]. The mechanism underlying these changes is inconclusive. Nitric oxide (NO) has been proposed as the physiological agent involved in this mechanism as it regulates feto-placental vascular permeability and resistance and platelet aggregation in the placenta [2–4]. Maturation and development of the placenta is affected significantly by epigenetics, [5] and therefore an epigenetic molecule such as nitric oxide has been postulated to affect foetal programming [6, 7] and foetal survival [8].

Various cell types in the human body produce nitric oxide largely via enzymatic pathways catalysed by nitric oxide synthase (with several iso-enzymes). Nitric oxide is synthesized from L-arginine, oxygen and tetrahydrobiopterin as a cofactor [9]. Endothelial nitric oxide produces vascular smooth muscle relaxation via its action through cyclic guanosine monophosphate dependent pathway [10, 11].

During normal foetal development, a proper vascular adaptation between the mother and the foetus is of crucial significance in regulating blood flow, nutrient supply and transport activities [12–14]. Nitric oxide has shown a beneficial effect in instances of abnormal vascular adaptation between the mother and the foetus, such as occurs in intrauterine growth retardation [15]. Nitric oxide has also been implicated in other abnormal feto-maternal vascular adaptations such as preeclampsia and gestational diabetes mellitus [16]. Nitric oxide mediates initiation of placental vasculogenesis and therefore embryonic stem cell differentiation towards the endothelial lineage through the effects of vascular endothelial growth factor (VEGF) [17, 18]. Some researchers have demonstrated that endothelial derived nitric oxide has a significant role in cardiovascular alterations in pregnancy [19].

Despite the proposed beneficial effects of nitric oxide in pregnancy, there is an inconclusive picture as far as serum levels of nitric oxide is concerned in normal pregnancy. For instance, some researchers noted that nitric oxide levels increase with gestation during normal pregnancy returning to non-pregnant levels some time post-partum [20–23]. In fact nitric oxide synthase expression has also been proved to progressively increase by some researches during normal pregnancy [24–26]. However, other researchers have reported varied opinions. Hata and colleagues reported decreasing serum nitric oxide levels as normal pregnancy progresses [27], whilst other investigators have also noted no change in nitric oxide levels during normal pregnancy [28, 29]. Therefore this study sought to find the serum nitric oxide levels among healthy pregnant women in a tertiary hospital in Ghana.

## Methods

This was a case-control study aimed at determining serum nitric oxide levels in women with healthy pregnancy using healthy non-pregnant women as controls at Korle-Bu Teaching Hospital, Accra, Ghana. The study was approved by the ethical and protocol review committee of the University of Ghana School of Medicine and Dentistry (Protocol Identification Number: CHS-Et/M.4-P4.5/2015-2016).

One hundred and thirty two (132) women aged 18-35 years comprising of 32 healthy non- pregnant women as controls and 100 healthy pregnant women (consisting of 33 first trimester [gestational age: 1-13 weeks], 37 s trimester [gestational age: 14-28 weeks] and 30 third trimester [gestational age: > 29 weeks]) as cases were sampled consecutively after fulfilling the inclusion criteria and obtaining an informed consent. Ultrasonography (done within the first trimester) and last menstrual period were used to determine the gestational age of the pregnant women. All recruited subjects carried singleton pregnancies and were on iron supplements.

Exclusion criteria included women who smoked, women with hypertension and/or diabetes, preeclamptics and women with acute and/or chronic infections.

The weight and height of recruited subjects were measured using mechanical patient weighing scale with height rod (product 6003, Inmoclinc, UK). The blood pressure of subjects was measured from the right arm using a mercury sphygmomanometer (nova-presameter®-Reister, Germany) and a stethoscope. Under aseptic conditions, 3mls of blood sample was obtained from the cubital vein of each recruited subject and kept in a sodium ethylenediamine tetraacetate (Na EDTA) test tube inverted severally to prevent clotting. The sample was then centrifuged at about 10,000 revolutions per minute (rpm) for 10 min to obtain the serum. The obtained serum was analysed for nitric oxide levels using the Greiss reagent method (Promega, Madison, USA). Determination of serum nitric oxide levels were done between the 10th – 12th week of gestation, 24th – 26th week of gestation and 35th – 36th week of gestation for first, second and third trimester patients respectively.

We chose to have three different groups at each trimester rather than follow the same patients because in a low income resource limited setting like ours, there is a high attrition rate from factors such as change in contact details of participants, refusal to participate in the course of a study, irregular antenatal attendance, inability of most first trimester pregnancies to reach term secondary to abortions and the high cost that may be associated with such a study design.

Unique codes were assigned to patients and electronic data were secured with passwords to ensure confidentiality. Age, weight, height, Body Mass Index (BMI), diastolic and systolic blood pressures, mean arterial blood pressure and serum nitric oxide levels measured were statistically analysed and reported as means (standard deviation) in Statistical Package for Social Sciences (SPSS) version 20. One-way Analysis of Variance (ANOVA) was used to compare statistically significant differences between the three groups of cases and the control and where a statistically significant difference was noted, a follow up test (post hoc analysis) was done. Parity among the various groups were compared using Kruskal-Wallis test.

## Results

There was no significant difference in the ages of women in the different study groups. There were however, significant differences among the studied groups with respect to the height, weight and BMI of subjects as shown in Table 1. The median (ranges) of parity of non-pregnant subjects was 1(0-5) whilst that for first, second and third trimester subjects were 1(1-2)**,** 1(0-3) and 1(0-7) respectively. There was no statistically significant difference in parity among the groups studied (*p*-value = 0.965).

**Table 1:** Demographic characteristics

	Non-pregnant	Trimester 1	Trimester 2	Trimester 3	*p-value*
Parameter	Mean (SD)	Mean (SD)	Mean (SD)	Mean (SD)	
*n*	32	33	37	30	
Age(years)	31.0(5.11)	29.1(3.76)	29.9(3.97)	29.9(2.60)	0.291
Weight(kg)	75.1(17.64)	63.7(11.14)	75.1(15.23)	76.8(14.21)	0.001*
Height(m)	1.62(0.05)	1.58(0.06)	1.65(0.07)	1.59(0.07)	<0.001*
BMI(kg/m^2^)	28.7(6.23)	24.7(6.25)	27.7(5.52)	30.5(5.50)	0.001*

*Significant *p* ≤ 0.05; n – sample size; *Mean (SD)* Mean (standard deviation); *BMI* body mass index

**Fig. 1:**
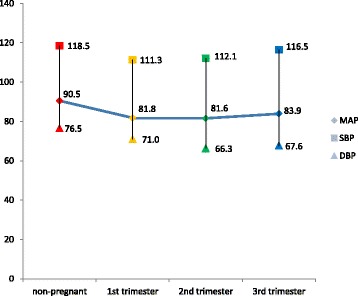
Trend of blood pressure changes in normal pregnancy. Y-axis: Mean blood pressure (mmHg). X-axis: Category of women. SBP: systolic blood pressure (*p* = 0.118). DBP: diastolic blood pressure (*p* < 0.001). MAP: mean arterial pressure (*p* = 0.009). The upper, lower and middle values represent mean systolic blood pressure, diastolic blood pressure and mean arterial pressure for category of women respectively

Although the systolic blood pressure did not significantly differ, the diastolic and mean arterial pressures significantly differed among the groups studied (Table 2 and Fig. 1). A follow-up test showed diastolic blood pressures to be significantly lower in the second and third trimesters compared to the non-pregnant state (*p* < 0.001 and *p* = 0.001 respectively). The mean arterial pressures were however, significantly lower in the first trimester (*p* = 0.020) and second trimester (*p* = 0.012) compared to the non-pregnant state. However, there was no significant inter-gestational change in diastolic and mean arterial pressure.

**Table 2:** Blood pressure and nitric oxide levels

	Nonpregnant	Trimester 1	Trimester 2	Trimester 3	*p-value*
Parameter	Mean(SD)	Mean(SD)	Mean(SD)	Mean (SD)	
*n*	32	33	37	30	
Systolic(mmHg)	118.5(16.08)	111.3(14.70)	112.1(12.11)	116.5(13.38)	0.118
Diastolic(mmHg)	76.5(9.35)	71.0(10.46)	66.27(7.68)	67.6(8.54)	<0.001*
MAP (mmHg)	90.5(9.71)	81.8(18.05)	81.6(8.55)	83.9(8.85)	0.009*
Serum Nitric Oxide (nM)	470.5(78.21)	1137.5(258.10)	1341.6(78.14)	1353.3(93.02)	<0.001*

*Significant p ≤ 0.05; n – sample size; *Mean (SD)* Mean (standard deviation)

**Fig. 2:**
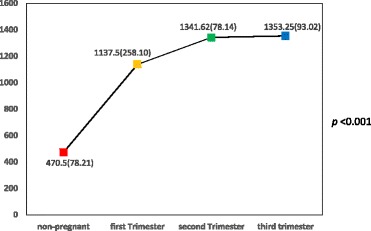
Trend of serum nitric oxide levels in normal pregnancy. Y-axis: Serum nitric oxide levels (nM). X-axis: Category of women

Serum nitric oxide levels among the various groups studied were found to be significantly different as shown in Table 2 and Fig. 2. A follow-up test showed serum nitric levels in the first trimester (*p* = 0 .010), second trimester (*p* < 0.001) and the third trimester (*p* < 0.001) to be significantly higher compared to the non-pregnant state. However, there was no significant inter-gestational change in serum nitric oxide levels.

## Discussion

The study revealed that healthy pregnancy results in a sudden significant increase in serum nitric oxide levels which progressively continues during pregnancy peaking in the third trimester (Fig. 2). The progressive increase in serum nitric oxide level with gestation is however not significant, a finding supported by other researchers [20–26]. Nitric oxide as a signalling molecule is involved in various processes including vascular tone regulation, cellular respiration, proliferation, apoptosis and gene expression. Trophoblastic invasion, placental development and placental vascular dilatation and thus placental blood flow are all mediated by nitric oxide produced locally as the placenta lacks both adrenergic and cholinergic innervation [13]. Nitric oxide may therefore play a vital role in foetal growth and sustenance as evidenced in this study by the progressive increase in its level during healthy pregnancy.

Other studies [27–29] however, found no progressive increase in serum nitric oxide levels during healthy pregnancy. The differences in findings may be due to differences in methods of nitric oxide measurement. Nitric oxide being very labile is usually measured by reactions that produce its metabolites. Incomplete reactions, differences in sensitivities and sample sources may account for the differences in results. Also serum nitric oxide levels are affected by differences in dietary intake and renal clearance. However, these factors were not noted or measured in this study.

From this study, there was a significantly reduced diastolic blood pressure (*p* < 0.001) and a significantly reduced mean arterial pressure (*p* = 0.009) in healthy pregnancy compared to the non-pregnant state similar to a report by Sanghavi and Rutherford [30]. Healthy pregnant women had a significant reduction in mean arterial pressure in first trimester (*p* = 0.020) and second trimester (*p* = 0.012) after which there was a slight but non-significant increase in mean arterial pressure (*p* = 0.132) compared to healthy non-pregnant women as observed in other studies [31, 32].

In this study there was a non-significant change in the inter-gestational mean arterial blood pressures among healthy pregnant women. Therefore the significant reduction in blood pressures occurs very early in pregnancy just as noted by Mahendru and colleagues [31].

It has been observed that during healthy pregnancy profound cardiovascular changes occur including peripheral vasodilatation leading to a reduction in arterial blood pressure [33]. Various chemical mediators have been implicated in this phenomenon including nitric oxide. Other mediators implicated in peripheral vasodilatation and therefore a reduction in blood pressure during healthy pregnancy are estradiol and prostacyclin [33].The finding of a significant drop in blood pressure in healthy pregnancy compared to the healthy non-pregnant state with a concomitant increase in serum nitric oxide may be due to nitric oxide mediated vasodilatation and reduction in systemic vascular resistance. Other mediators implicated in vascular changes during healthy pregnancy were however not determined in this study.

Serum nitric oxide levels mimics variations in blood pressures during normal pregnancy and it is thus possible that dysfunctions in nitric oxide production and release may play a role in disorders of abnormal vascular function in pregnancy and foetal well-being.

The human feto-placental circulation exhibits a low vascular resistance. Thus circulating and locally released vasoactive molecules such as nitric oxide are likely to be involved in the control of feto-placental haemodynamics and therefore critical in foetal nutrition and oxygenation. Nitric oxide also causes inhibition of platelet aggregation thereby improving the feto-maternal circulation.

It may therefore be worthwhile exploring the use of exogenous nitric oxide sources in the management of conditions associated with abnormal vascular function in pregnancy so as to improve maternal and foetal outcomes.

## Conclusions

The finding of a significantly reduced blood pressures and a significant increase in serum nitric oxide levels in healthy pregnancy may suggest a role of nitric oxide in vascular adaptation in pregnancy.

## Abbreviations

ANOVA: Analysis of Variance
BMI: Body Mass Index
mls: Millilitres
Na EDTA: Sodium ethylenediamine tetraacetate
rpm: Revolution per minute
VEGF: Vascular endothelial growth factor
